# Necrotizing Soft Tissue Infection of Left Shoulder and Upper Limb Following Intravenous Injection of Non-steroidal Anti-inflammatory Drug

**DOI:** 10.7759/cureus.18068

**Published:** 2021-09-17

**Authors:** Elaine Zi Fan Soh

**Affiliations:** 1 Orthopaedics, Hospital Segamat, Johor, MYS

**Keywords:** non-steroidal anti-inflammatory drug, diabetic, intravenous injection, klebsiella, necrotizing soft tissue infection

## Abstract

Necrotizing soft tissue infection (NSTI) is a rapidly spreading and fulminant infection that may occur within any layer of skin and soft tissue and may result in sepsis and multiorgan failure. Cases of intravenous injection of drugs causing NSTIs have been reported, especially involving injection of illicit drugs or insulin. NSTI can be due to polymicrobial or monomicrobial infection and infection involving Klebsiella species has been rising, especially in patients with diabetes mellitus. This is a case of an extensive upper limb and shoulder *Klebsiella pneumoniae* NSTI following an injection of a non-steroidal anti-inflammatory drug in a diabetic patient. NSTI diagnosis is based on history and clinical examination, supplemented with imaging and laboratory investigations. Early recognition, extensive and serial debridement, antibiotics, and wound management are crucial for the better outcome of the disease. This patient underwent emergent debridement with antibiotics coverage, followed by serial debridement and wound care. The wound healed within the stipulated time, with good function of the affected limb following the rehabilitation program.

## Introduction

Necrotizing soft tissue infection (NSTI) is a fulminant infection that may occur within any layer of the skin and soft tissue. It is characterized by the presence of toxin-producing bacteria, local tissue destruction, fulminant progression of the inflammatory process, and early systemic toxicity that may result in sepsis, multi-organ failure, and death [1]. Soft tissue infection resulting from subcutaneous, intravenous, or intramuscular injection with contaminated needles may present with a wide range of severities, including cellulitis, abscess, myositis, and necrotizing fasciitis. The organisms involved are usually polymicrobial most commonly *Staphylococcus aureus* and *Streptococcus pyogenes* [2]. Monomicrobial necrotizing fasciitis caused by *Klebsiella pneumoniae* is now an emerging infectious disease, especially among those with diabetes mellitus [3]. This article highlights a presentation of *Klebsiella pneumoniae* NSTI over the left arm and shoulder following intramuscular injection of a non-steroidal anti-inflammatory drug. 

## Case presentation

**Figure 1:**
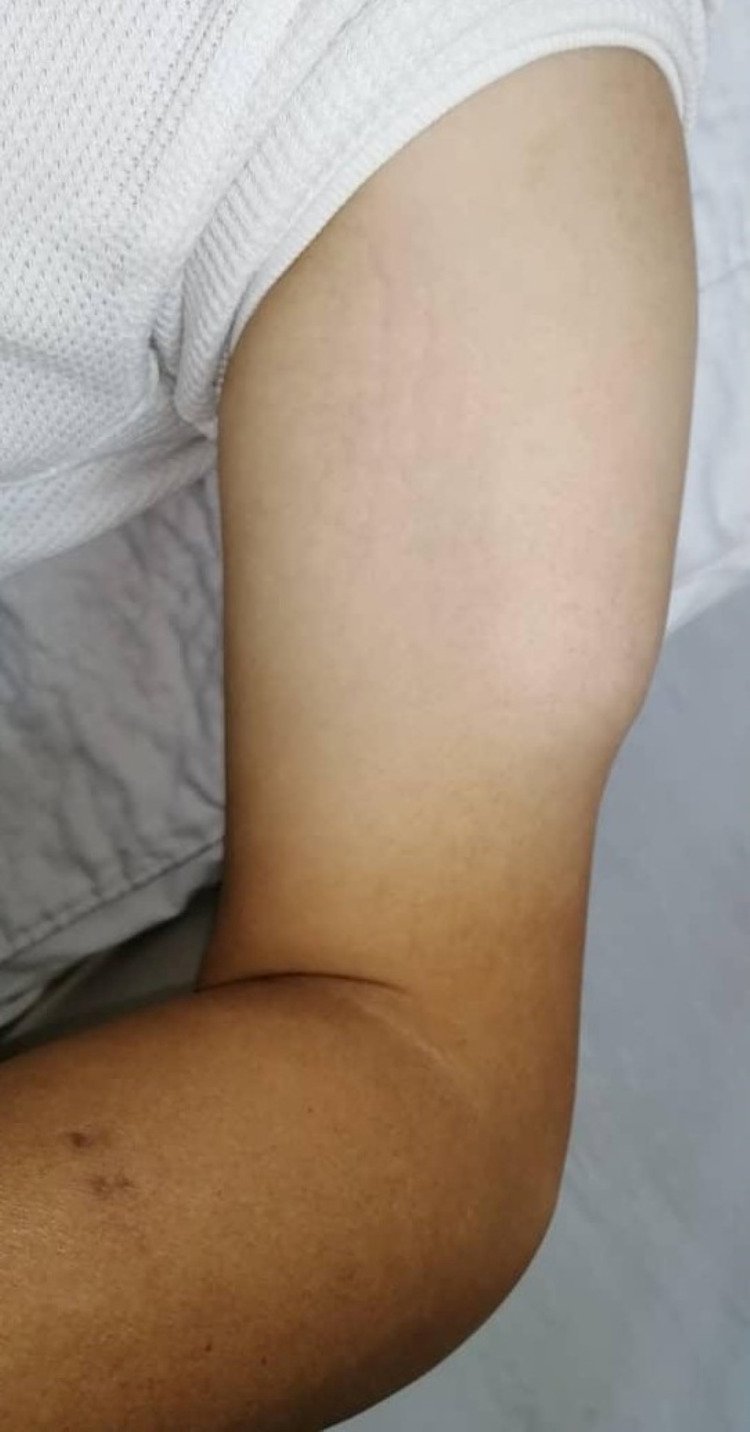
Diffuse swelling over left deltoid region extending towards distal arm with poor demarcation border

A 37-year-old male, recently diagnosed with uncontrolled type II diabetes mellitus on metformin, presented with swelling and pain over the left deltoid region for the past five days, associated with fever. Further history revealed that he received an injection of a non-steroidal anti-inflammatory drug (diclofenac) intramuscularly over left deltoid region five days ago, at a general practitioner clinic for his three days history of flu-like symptoms. On presentation, he was noted to be feverish with a temperature of 38 °C. This was accompanied by a diffuse swelling over left deltoid region extending towards the distal arm with a poor demarcation border, and crepitus felt (Figure 1). There were no obvious skin erythematous changes or any skin lesion or bullae formation.

**Figure 2:**
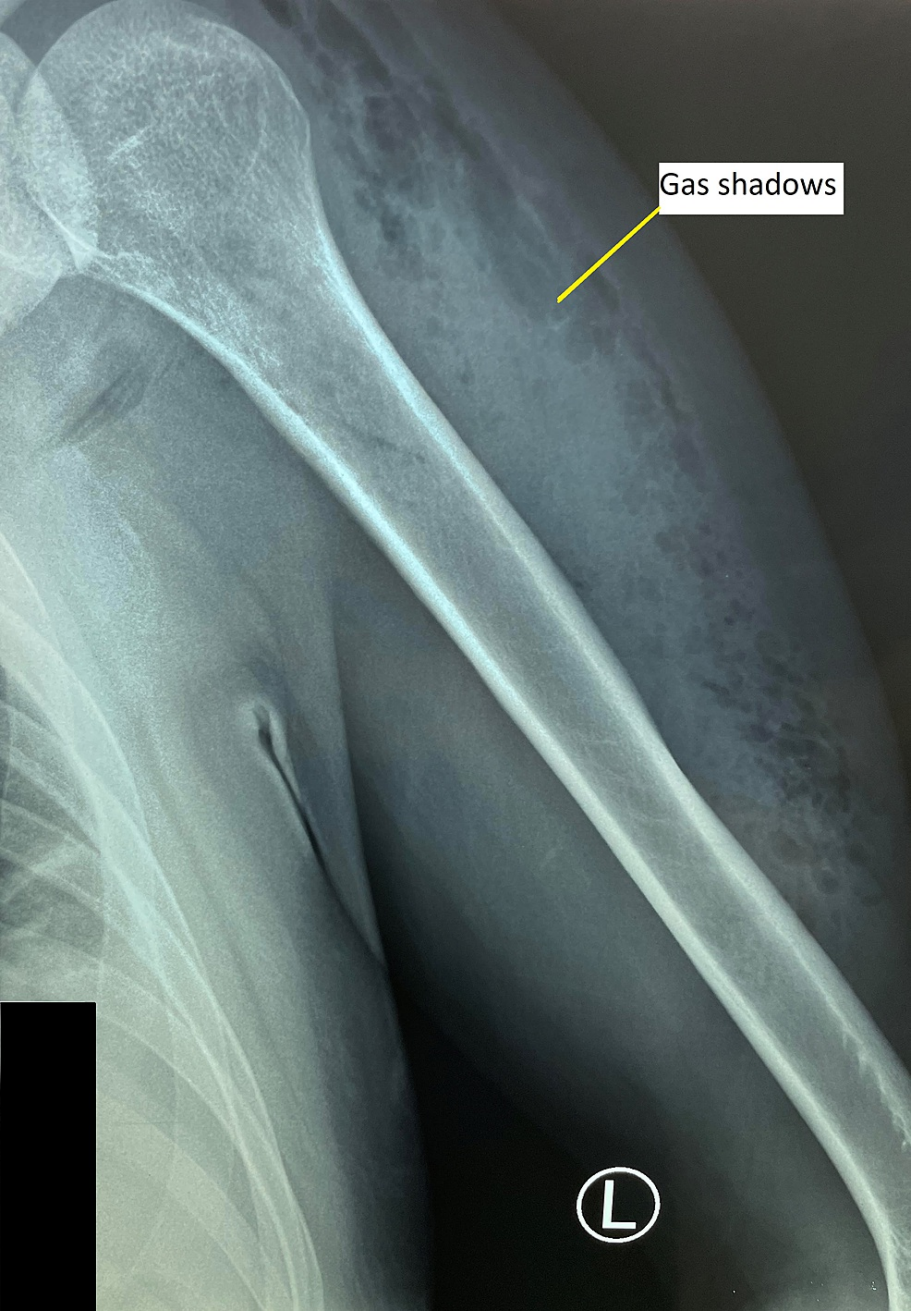
Plain radiography of the left humerus showed presence of gas shadow over the left deltoid region extending along the lateral and posterior aspect of left arm.

Plain radiography of the left humerus showed the presence of gas shadow over the left deltoid region extending along the lateral and posterior aspect of the left arm. There was no evidence of osteomyelitis changes over the humerus or periosteal reaction (Figure 2). His blood tests showed a raised white blood cells (28.02 X 10 3/uL) with neutrophil predominance and C-reactive protein of 48.26mg/dL.

**Figure 3:**
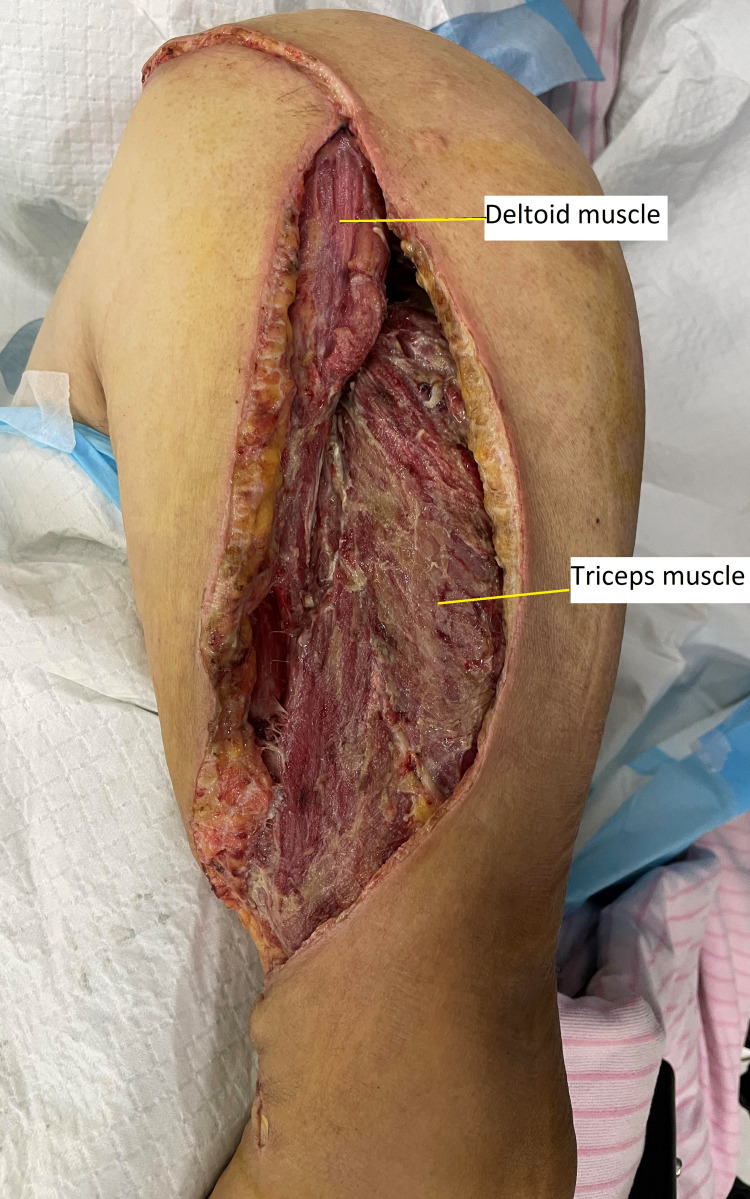
Figure showing debridement done through lateral aspect of deltoid, with unhealthy subcutaneous tissue and fascia with pus drained from intramuscular from lateral and posterior head of deltoid and triceps muscle

Initial clinical suspicion was indicative of NSTI. An emergency debridement was performed through an incision over the lateral aspect of the deltoid. There were unhealthy subcutaneous tissue and fascia, with pus drained from intramuscular from the lateral and posterior head of deltoid and triceps muscles. Affected muscles that appeared diseased were also debrided (Figure 3). He was started on intravenous piperacillin-tazobactam. Tissue and pus culture and sensitivity test grew *Klebsiella pneumoniae,* which had sensitivity towards the antibiotic choice.

**Figure 4:**
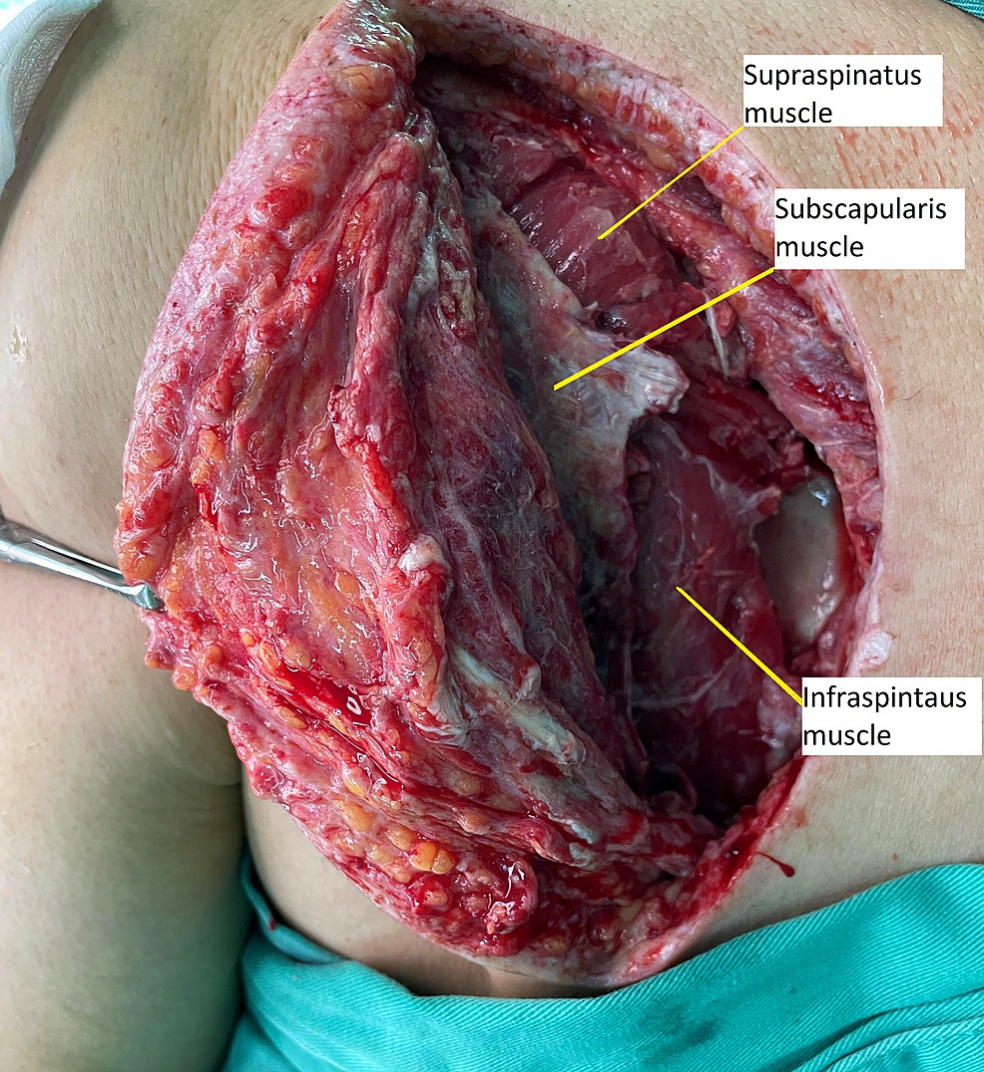
Figure showing the pus tracking posterior to deltoid muscle and along the infraspinatus muscle and unhealthy muscles debrided

Few days later, there was still persistent pus tracking from the posterior aspect of deltoid. CT of the left shoulder and arm was performed and showed there is a collection in the scapular region and over the anterior shoulder region. He underwent another debridement with a separate incision along the scapular spine towards the medial border of the scapula. There was pus tracking posterior to deltoid muscle and along the infraspinatus muscle (Figure 4). The previous incision was extended proximally towards the shoulder using a deltopectoral approach but noted muscles were healthy. Aspiration from the left shoulder joint also showed no pus. The repeated sample from the operation also showed *Klebsiella pneumoniae.*

The patient subsequently also developed an intramuscular abscess over the left calf that required incision and drainage and the sample yielded the same organism. He required multiple wound debridements over the arm and scapula region before a secondary closure was done. He received a total course of two weeks of piperacillin-tazobactam intravenously, and subsequently was changed to ampicillin-sulbactam for another two weeks until his wound condition improved.

The patient was cleared for discharge after a month-long stay in the hospital. His blood parameters showed remarkable improvement with white blood cell counts of 9.36 X 10 3/uL and C-reactive protein of 5.02mg/dL. The patient was followed up with regular physiotherapy sessions for the shoulder range of motion and muscle strengthening. On two months follow-up in the clinic, the patient was well, remained afebrile, and wound was well healed. He achieved a good range of motion of shoulder and adequate arm strength and able to return to his work and daily activities.

## Discussion

NSTIs can occur in anywhere in the body but are most commonly found in the extremities, perineum, and genitalia, with some arising on the chest or the abdomen. It usually occurs through any break in the epithelial or mucosal surface, resulting in the inoculation of the organism into the subcutaneous tissue. Multiple etiologies reported include trauma, intravenous drug and insulin injection, skin infections and ulcers, animal and insect bites, visceral-cutaneous fistulas, surgical complications, percutaneous catheter insertion, abscesses, and idiopathic etiologies [4].

There were some similar cases reported in the literature on NSTIs after intravenous injection. Ginting et al. reported a similar case of upper extremity NSTI in an intravenous drug user after injecting with cocaine mixed with flavored drink mix requiring serial wound debridement and reconstruction [5].

NSTI can be due to multiple pathogens either polymicrobial or monomicrobial. *Klebsiella pneumoniae* NSTI is seen as an emerging infectious disease worldwide and has a higher rate of hematogenous spread and potential for metastatic infection than other pathogens. Diabetis mellitus is the most common host characteristic associated with this infection [3]. *Klebsiella pneumoniae* infection may be associated with abscesses in other locations in the body such as a hepatic abscess or calf abscess as seen in our case [3].

The standard treatment for NSTI comprises broad-spectrum antibiotics and wide surgical debridement and supportive care. It has significant morbidity and mortality rates of 25-35% [4]. Hence, stressing the importance of rapid and serial surgical debridement, rapid initiation of antibacterial agents and proper wound management is essential to improve the outcome of the patient [1]. 

## Conclusions

NSTI is a rapidly progressive and fulminant disease. Early recognition, prompt treatment, extensive debridement with the appropriate antibiotic is the key to a favorable outcome.
